# Epitranscriptomic control of epithelial–mesenchymal transition in cancer: mechanisms, plasticity, and therapeutic opportunities

**DOI:** 10.3389/fcell.2026.1890102

**Published:** 2026-07-01

**Authors:** Juan Luo, Xiulin Jiang, Chongxin Li

**Affiliations:** 1 Department of Oncology, Qujing Hospital of Traditional Chinese Medicine, Qujing, China; 2 College of Life Science, University of Chinese Academy of Sciences, Beijing, China; 3 Department of Radiotherapy, Qujing Central Hospital of Yunnan Province / Kunming Medical University Affiliated Qujing Hospital, Qujing, China

**Keywords:** cancer invasion, cell migration, EMT, epitranscriptomics, HIF-1α, hypoxia, immune evasion, RNA modification

## Abstract

Epithelial–mesenchymal transition (EMT) is a flexible cell-state program that supports tumor invasion, metastasis, immune escape, and therapy resistance. It is not a simple switch from an epithelial to a mesenchymal phenotype. Instead, cancer cells often move through intermediate or partial EMT states, which allow them to retain cell–cell adhesion while gaining motility and stress tolerance. Recent studies show that RNA modifications, including N6-methyladenosine (m^6^A), 5-methylcytosine (m^5^C), N1-methyladenosine (m^1^A), A-to-I RNA editing, pseudouridine (Ψ), N4-acetylcytidine (ac^4^C), and N7-methylguanosine (m^7^G), add an important post-transcriptional layer to EMT regulation. These modifications control RNA stability, translation, splicing, export, and innate immune sensing. They therefore connect environmental cues, such as hypoxia, TGF-β signaling, inflammatory cytokines, and therapeutic stress, to EMT-related gene expression programs. This review summarizes how major RNA modification systems regulate EMT in cancer. Rather than listing individual findings, we compare common regulatory patterns across tumor types. m^6^A has the strongest evidence base and acts through writer–reader–eraser modules that regulate EMT transcription factors and signaling pathways such as TGF-β/SMAD, Wnt/β-catenin, PI3K/AKT, EGFR/STAT3, and Notch. m5C and ac^4^C mainly promote EMT by stabilizing transcripts and enhancing translation, whereas m7G influences EMT through translational reprogramming and codon-biased protein synthesis. A-to-I editing has more complex effects because it can either support immune evasion and plasticity or generate tumor-suppressive RNA isoforms. Ψ-related mechanisms remain less developed, but early evidence suggests roles in RNA stability, stress adaptation, and invasive behavior. We also discuss how EMT and RNA modifications interact with the tumor microenvironment, especially immune suppression and checkpoint resistance. Finally, we evaluate therapeutic opportunities and key challenges. Current studies are limited by reliance on bulk assays, incomplete site-specific validation, weak causal evidence, and insufficient clinical standardization. Future work should integrate single-cell and spatial epitranscriptomics, functional RNA editing tools, and clinical cohorts to define which RNA modification events are true drivers of EMT and which are only associated markers.

## 1 Introduction

Epithelial–mesenchymal transition (EMT) is a major program of tumor plasticity. During EMT, epithelial cancer cells lose polarity and cell–cell adhesion and gain migratory and invasive properties (Lamouille et al., 2014). This process contributes to local invasion, distant metastasis, drug resistance, radiotherapy resistance, and cancer stemness (Lamouille et al., 2014). For this reason, EMT has long been viewed as a central mechanism of tumor progression and treatment failure. However, the classical view of EMT as a complete conversion from an epithelial state to a mesenchymal state is now considered too simple. In many tumors, cancer cells do not fully complete EMT. Instead, they adopt partial EMT states, in which epithelial and mesenchymal features coexist (Pastushenko and Blanpain, 2019). These hybrid states may be especially important for metastasis because they allow cells to migrate while maintaining collective behavior and survival signals. EMT is also reversible. Mesenchymal-like tumor cells can undergo mesenchymal-to-epithelial transition (MET), especially during metastatic colonization. Thus, EMT should be understood as a dynamic spectrum rather than a fixed endpoint. Traditional EMT research has focused on transcription factors such as SNAIL, SLUG, TWIST, and ZEB, as well as signaling pathways including TGF-β, Wnt/β-catenin, Notch, and hypoxia-related signaling (Pastushenko and Blanpain, 2019). Epigenetic mechanisms, including DNA methylation, histone modification, and chromatin remodeling, also shape EMT by controlling chromatin accessibility and transcriptional output (Pastushenko and Blanpain, 2019). These layers explain how EMT genes are turned on or off, but they do not fully explain the rapid and reversible nature of EMT under stress.

RNA modifications provide an additional explanation. Epitranscriptomic marks, including m^6^A, m5C, m1A, A-to-I editing, Ψ, ac4C, and m7G, can rapidly alter RNA stability, translation, splicing, localization, and immune recognition (Einstein et al., 2021; Chen X. et al., 2024). Because these modifications act after transcription, they allow tumor cells to quickly adjust protein production without changing DNA sequence or chromatin state. This makes RNA modifications well suited to regulate EMT, which is highly responsive to hypoxia, inflammation, cytokines, metabolic stress, and therapy (Zhang W. et al., 2024).

This review discusses how RNA modifications regulate EMT in cancer. We focus on shared mechanisms, differences among RNA modification types, and the strength of current evidence. We also highlight unresolved questions, including context dependence, causality, tumor heterogeneity, and therapeutic feasibility.

## 2 Overview of RNA modifications in cancer

**FIGURE 1:**
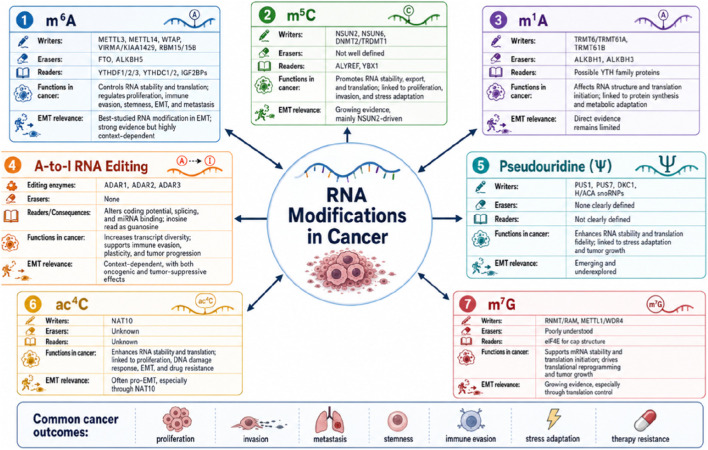
Overview of major RNA modifications involved in cancer and EMT-related phenotypes. This figure summarizes seven major RNA modification systems implicated in cancer biology, including N6-methyladenosine (m^6^A), 5-methylcytosine (m^5^C), N1-methyladenosine (m^1^A), adenosine-to-inosine (A-to-I) RNA editing, pseudouridine (Ψ), N4-acetylcytidine (ac^4^C), and 7-methylguanosine (m^7^G). For each modification, representative writers, erasers, readers, major cancer-related functions, and EMT relevance are shown. The key message is that m^6^A currently has the strongest mechanistic evidence in EMT regulation, whereas m^5^C, A-to-I editing, ac^4^C, m^7^G, m^1^A, and Ψ show emerging or context-dependent evidence. Together, these modifications influence common cancer outcomes, including proliferation, invasion, metastasis, stemness, immune evasion, stress adaptation, and therapy resistance. Abbreviations: EMT, epithelial–mesenchymal transition; m^6^A, N6-methyladenosine; m^5^C, 5-methylcytosine; m^1^A, N1-methyladenosine; Ψ, pseudouridine; ac^4^C, N4-acetylcytidine; m^7^G, 7-methylguanosine.

To provide a general framework, we first summarize the major RNA modification types involved in cancer biology and compare their regulatory enzymes, molecular functions, and current evidence in EMT (Figure 1).

### 2.1 m^6^A

m^6^A is the most widely studied internal modification in messenger RNA. It is enriched near stop codons, in 3′untranslated regions, and in some intronic regions (Liu et al., 2014). Its regulation depends on a writer–eraser–reader system. The core writer complex contains METTL3 and METTL14, together with cofactors such as WTAP, KIAA1429/VIRMA, and RBM15/15B (Wei et al., 2022; Dou et al., 2023). FTO and ALKBH5 remove m^6^A marks, whereas YTH domain proteins and IGF2BP family proteins recognize m^6^A-modified transcripts (Chen X. et al., 2025; Weng et al., 2022; Xiao et al., 2024; Liu C. et al., 2024; Chen et al., 2023). Functionally, m^6^A can promote either RNA decay or RNA stabilization depending on the reader protein involved. For example, YTHDF2 is often linked to mRNA degradation, while IGF2BPs usually stabilize transcripts (Chen Z. et al., 2025). This explains why the same writer enzyme can have opposite biological effects in different tumors (Chen et al., 2023). In cancer, m^6^A regulates proliferation, immune evasion, stemness, EMT, and metastasis (Fan et al., 2024). Among all RNA modifications, m^6^A has the strongest mechanistic link to EMT, but it also shows the clearest context dependence.

### 2.2 m^5^C

m^5^C occurs in mRNA, tRNA, and rRNA (Cai et al., 2025). Its main writers include NSUN family proteins, especially NSUN2 and NSUN6, and DNMT2/TRDMT1 (Hu et al., 2021). ALYREF and YBX1 have been reported as reader proteins that regulate mRNA export, stability, and translation (Wang et al., 2023). In cancer, m^5^C often supports proliferation, invasion, stress adaptation, and metastasis (Li R. et al., 2024). Compared with m^6^A, m^5^C research in EMT is less mature. Many studies focus on NSUN2 and show that it promotes tumor progression by stabilizing oncogenic transcripts. However, NSUN6 may have tumor-suppressive effects in some settings. Thus, m^5^C should not be treated as uniformly oncogenic. The biological outcome depends on the writer, target transcript, reader protein, and tumor context.

### 2.3 m^1^A

m1A is a positively charged adenine modification found mainly in tRNA, rRNA, and the 5′UTR of some mRNAs (Miao et al., 2025). TRMT6/TRMT61A and TRMT61B act as writers, while ALKBH1 and ALKBH3 can remove m1A marks (Zhong et al., 2024; Gu et al., 2024). m1A can affect RNA structure and translation initiation (Li Y. et al., 2026). In cancer, m1A has been associated with increased protein synthesis, cell growth, and metabolic adaptation (Tao et al., 2025). However, direct evidence linking m1A to EMT remains limited. At present, m1A should be viewed as a candidate regulator of EMT rather than a well-established EMT driver.

### 2.4 A-to-I RNA editing

A-to-I RNA editing is mediated by ADAR family enzymes, especially ADAR1 in cancer (Liang et al., 2024). It converts adenosine to inosine, which is read as guanosine during translation or RNA processing (Nishikura, 2016). This process can alter coding sequences, splicing, microRNA binding, and innate immune recognition. ADAR1 is frequently upregulated in tumors and is associated with immune evasion, progression, and plasticity (Shen et al., 2021). A-to-I editing is different from methylation-based RNA modifications because it can change RNA sequence information. It can also suppress double-stranded RNA sensing, helping cancer cells avoid innate immune activation (Fan et al., 2024; Cai et al., 2025). Therefore, A-to-I editing may regulate EMT through both gene-expression remodeling and immune escape.

### 2.5 Pseudouridine (Ψ)

Pseudouridine (Ψ) is the most abundant RNA isomerization modification (Niu and Liu, 2023). It is catalyzed by pseudouridine synthases such as PUS1, PUS7, and DKC1, as well as H/ACA snoRNP complexes (Stockert et al., 2021). Ψ can increase RNA stability, improve base pairing, and enhance translation fidelity (Yang et al., 2025). In cancer, Ψ-related pathways have been linked to protein synthesis, stress adaptation, and tumor growth (Yang et al., 2025). However, direct evidence in EMT is still sparse. Current studies suggest that Ψ may influence EMT by regulating mRNA stability and stress-response pathways, but the field still lacks systematic mapping and functional validation.

### 2.6 ac^4^C

ac4C is an RNA acetylation mark found in rRNA, tRNA, and mRNA (Xiong et al., 2026). NAT10 is the only well-confirmed writer enzyme. Dedicated erasers and readers have not been clearly defined. ac4C can increase RNA stability and translation efficiency (Li C. et al., 2025). In cancer, NAT10-mediated ac4C is linked to proliferation, DNA damage response, metastasis, and therapy resistance (Xiong et al., 2026). A recurring pattern is that NAT10 stabilizes transcripts involved in TGF-β signaling, cytoskeletal remodeling, or EMT marker expression. This makes NAT10 a potentially targetable node, although the lack of defined readers and erasers limits mechanistic precision. It should also be noted that NAT10 is a multifunctional protein and its biological effects should not be automatically attributed solely to ac4C modification (Zhang S. et al., 2024). In addition to catalyzing ac4C formation on RNA, NAT10 has been reported to participate in ribosome biogenesis, nuclear architecture, chromatin regulation, and other cellular processes (Liu X. et al., 2018; Larrieu et al., 2014; Amin et al., 2025). Therefore, phenotypes observed after NAT10 knockdown, knockout, or pharmacological inhibition may reflect both ac4C-dependent and ac4C-independent mechanisms. Unless site-specific ac4C changes are directly validated, NAT10-associated effects on EMT should be interpreted with caution and should not be simply defined as ac4C-dependent regulation.

### 2.7 m^7^G

m^7^G is best known as the 5′cap modification of eukaryotic mRNA, but it is also present in tRNA, rRNA, and internal mRNA sitesA (Wang et al., 2025). RNMT/RAM regulates mRNA cap methylation, while METTL1–WDR4 mainly catalyzes m^7^G in tRNA and some internal RNA sites (Gu et al., 2024). The m^7^G cap is recognized by translation initiation factors such as eIF4E and is essential for mRNA stability and translation initiation (Wang et al., 2025). In cancer, m^7^G supports translational capacity and tumor growth. METTL1-mediated tRNA m^7^G can reshape codon-specific translation and favor the production of proteins required for rapid proliferation and malignant progression (Wang et al., 2025; Zhang J. et al., 2026). This mechanism is relevant to EMT because EMT requires coordinated translation of signaling molecules, cytoskeletal proteins, and transcription factors.

## 3 EMT program in cancer

### 3.1 EMT transcription factors

The core EMT transcription factors include SNAIL/SNAI1, SLUG/SNAI2, TWIST1/2, and ZEB1/2 (Saitoh, 2023). These factors repress epithelial genes such as CDH1/E-cadherin and activate mesenchymal programs (Saitoh, 2023). SNAIL and SLUG often act as early response factors induced by TGF-β, Wnt, inflammatory signals, and hypoxia. TWIST proteins support migration and resistance to apoptosis (Stemmler et al., 2019). ZEB1 and ZEB2 are important for maintaining mesenchymal features and cancer stemness (Debnath et al., 2022). Although these transcription factors are often discussed separately, they rarely act alone. They form interconnected circuits with microRNAs, chromatin regulators, RNA-binding proteins, and signaling pathways. Therefore, EMT is best viewed as a network state rather than the result of one master regulator.

### 3.2 Molecular hallmarks

Classical EMT hallmarks include loss of E-cadherin, gain of N-cadherin and vimentin, cytoskeletal remodeling, loss of epithelial polarity, and acquisition of spindle-like morphology (Voutsadakis, 2023). These changes increase migration and invasion. However, relying only on a few markers can be misleading. E-cadherin loss or vimentin gain does not always prove functional EMT. Some tumors metastasize through collective invasion while retaining epithelial markers. Others show partial EMT without complete cadherin switching. Therefore, EMT assessment should combine marker expression, cell-state profiling, functional invasion assays, lineage tracing, and ideally single-cell analysis.

### 3.3 EMT plasticity

EMT is reversible and highly plastic (Bakir et al., 2020). Partial EMT is particularly relevant to metastasis because hybrid epithelial/mesenchymal cells can retain adhesion while gaining mobility (Lüönd et al., 2021). MET may then support metastatic outgrowth after disseminated cells arrive at distant organs. These transitions create intratumoral heterogeneity and help cancer cells adapt to immune attack, drug treatment, and environmental stress (Aiello et al., 2018). RNA modifications are well positioned to regulate this plasticity. Unlike DNA mutations, RNA marks are reversible. Unlike transcriptional programs, they can alter protein output rapidly. Figure 1 summarizes how RNA modifications, including m^6^A, m^5^C, ac4C, m^7^G, and Ψ, may coordinate EMT-related signaling pathways such as TGF-β/SMAD, PI3K/AKT, Wnt/β-catenin, Notch, and EGFR across tumor types. Epitranscriptomic RNA modifications do not act as isolated marks but form interconnected and combinatorial regulatory networks that coordinate RNA folding, stability, translation, stress responses, transcript fate, and disease-related cellular states (Eladl et al., 2026).

## 4 RNA modifications regulate EMT: molecular mechanisms

**FIGURE 2:**
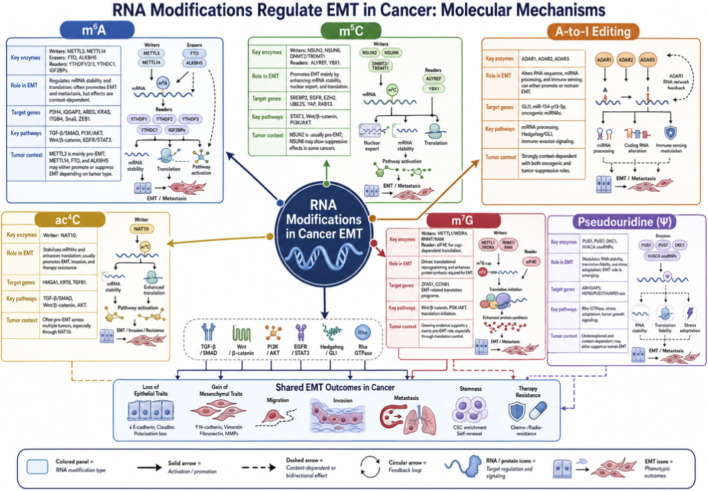
Molecular mechanisms by which RNA modifications regulate EMT in cancer. This figure illustrates how different RNA modification pathways regulate EMT-related gene expression through effects on RNA stability, translation, splicing, RNA editing, and signaling pathway activation. m^6^A, m^5^C, A-to-I editing, ac^4^C, m^7^G, and Ψ regulate EMT through distinct but partially overlapping mechanisms involving target transcripts, reader proteins, and downstream signaling pathways such as TGF-β/SMAD, Wnt/β-catenin, PI3K/AKT, EGFR/STAT3, Hedgehog/GLI, and Rho GTPase signaling. The key message is that RNA modifications do not act as simple pro- or anti-EMT switches; instead, their effects depend on the modified transcript, available reader proteins, tumor context, and downstream signaling environment. Solid arrows indicate activation or promotion, dashed arrows indicate context-dependent or bidirectional effects, and circular arrows indicate feedback loops. Abbreviations: EMT, epithelial–mesenchymal transition; TGF-β, transforming growth factor-beta; EGFR, epidermal growth factor receptor; STAT3, signal transducer and activator of transcription 3; PI3K, phosphoinositide 3-kinase; AKT, protein kinase B; CSC, cancer stem cell.

Figure 2 illustrates how RNA modifications and A-to-I RNA editing regulate EMT by controlling RNA fate. Writers, erasers, and readers can change the abundance, translation, localization, or immune visibility of EMT-related transcripts. These effects influence epithelial markers, mesenchymal markers, partial EMT states, and MET. To provide a more accessible overview of the evidence discussed in the text, we added Table 1, which summarizes individual RNA modification regulators, their functional categories, cancer contexts, EMT-related mechanisms, evidence levels, therapeutic relevance, and representative references.

**TABLE 1:** Detailed summary of RNA modification regulators involved in EMT regulation in cancer.

RNA modification	Regulator	Functional	Cancer type	Mechanism	Evidence level	Therapeutic relevance	References
m^6^A	METTL3	Writer	Bladder cancer	Stabilizes P3H4 mRNA, promoting proliferation, migration, and EMT	Cell-line/molecular evidence	Potential pro-EMT therapeutic target	Liu C. et al. (2024)
m^6^A	METTL3/IGF2BP2	Writer–reader axis	Hepatocellular carcinoma	m^6^A modification of IQGAP3 mRNA; IGF2BP2 increases transcript stability and activates TGF-β/SMAD signaling	Cell-line and mechanistic evidence	May predict metastasis risk; potential targetable m^6^A–reader axis	Pan et al. (2025)
m^6^A	METTL3	Writer	Pancreatic cancer	Methylates and stabilizes AREG mRNA, promoting EMT; negatively regulated by miR-33a-3p	Cell-line and mechanistic evidence	Potential target for EMT and metastasis inhibition	Su et al. (2023)
m^6^A	METTL3/IGF2BP3	Writer–reader axis	Bladder cancer	Stabilizes circPSMA7, regulating the miR-128-3p/MAPK1 pathway and promoting EMT-related progression	Cell-line and molecular evidence	May affect tumor progression and EMT plasticity	Yi et al. (2024)
m^6^A	METTL3	Writer	Cervical cancer	m^6^A modification of KRAS enhances interaction with SMAD2/3 and SNAIL, activating TGF-β/SMAD/SNAIL signaling	Cell-line and mechanistic evidence	Potential target for EMT and extracellular matrix remodeling	Gong et al. (2026)
m^6^A	METTL3	Writer	Proliferative vitreoretinopathy	METTL3 overexpression suppresses TGF-β1-induced EMT, suggesting anti-EMT function in a non-tumor fibrotic context	Disease model evidence	Highlights context-dependent effects; caution for therapeutic targeting	Liu C. et al. (2024)
m^6^A	METTL14/YTHDF2	Writer–reader axis	Bladder cancer	Suppresses migration and invasion by regulating USP38 through YTHDF2-mediated RNA decay	Cell-line and mechanistic evidence	Potential EMT-suppressive biomarker	Huang et al. (2022)
m^6^A	METTL14	Writer	Clear cell renal cell carcinoma	Promotes degradation of ITGB4 mRNA, inhibits PI3K/AKT signaling, and suppresses EMT	Cell-line and molecular evidence	Potential tumor-suppressive regulator	Liu H. et al. (2022)
m^6^A	METTL14	Writer	Cervical cancer	Methylates HOXB13 and activates NF-κB signaling, promoting EMT	Cell-line and mechanistic evidence	Demonstrates context-dependent oncogenic function	Li Q. et al. (2024)
m^6^A	RBM15/IGF2BP3	Non-canonical writer/reader axis	Gastric cancer	Regulates m^6^A modification of ECT2 mRNA through IGF2BP3 recognition; suppresses EMT and enhances chemotherapy sensitivity	Cell-line and mechanistic evidence	May improve chemosensitivity	Zhu et al. (2026)
m^6^A	RBM15	Non-canonical writer	Cervical cancer	Stabilizes lncRNA HEIH, activating the miR-802/EGFR axis and promoting EMT	Cell-line and mechanistic evidence	Potential EMT-associated therapeutic target	Quan et al. (2024)
m^6^A	METTL16	Non-canonical writer	Multiple cancer contexts	Regulates EMT indirectly through targets such as FBXO5 and SYNPO2L, affecting PI3K/AKT signaling and tumor microenvironment remodeling	Mainly mechanistic/bioinformatic evidence	Emerging target; requires further validation	Wang et al. (2024), Wu J. et al. (2024)
m^6^A	ALKBH5	Eraser	NSCLC	Demethylates TGFβR2 and SMAD3 transcripts, reducing their stability and suppressing TGF-β/SMAD-driven EMT	Cell-line and mechanistic evidence	Potential EMT-suppressive regulator	Sun et al. (2022)
m^6^A	ALKBH5	Eraser	Cisplatin-resistant cancer models	Regulates CEMIP mRNA stability, suppresses EMT, and enhances drug sensitivity	Drug-resistance cell model	May restore cisplatin sensitivity	Gao et al. (2024)
m^6^A	CREB/ALKBH5/SNAIL	Transcriptional regulator–eraser axis	Ovarian cancer	FSH activates CREB/ALKBH5, reducing m^6^A on SNAIL mRNA, increasing SNAIL stability and promoting EMT	Cell-line and mechanistic evidence	Relevant to hormone-associated EMT and metastasis	Xu et al. (2024)
m^6^A	ALKBH5	Eraser	Cancer stem cells of NSCLC	Supports stemness; ALKBH5 knockdown increases E-cadherin and reduces Nanog/Oct4, suppressing EMT-like phenotypes	Cancer stemness/cell-line evidence	Potential target for EMT-linked stemness	Liu X. et al. (2022)
m^6^A	FTO	Eraser	Thyroid cancer	Demethylates CDH12 mRNA, blocks IGF2BP2-mediated stabilization, and suppresses EMT	Cell-line and mechanistic evidence	Potential EMT-suppressive regulator	Chen Z. et al. (2024)
m^6^A	FTO	Eraser	Prostate cancer	FTO knockdown enhances EMT and migration, whereas FTO restoration reverses these effects	Cell-line evidence	Context-dependent therapeutic consideration	Zhao et al. (2024)
m^6^A	FTO	Eraser	Pancreatic cancer	FTO overexpression promotes proliferation, migration, invasion, and EMT	Cell-line evidence	FTO inhibition may suppress EMT in selected cancers	Ou et al. (2022)
m^6^A	FTO/ZEB1	Eraser–target axis	Breast cancer	Stabilizes ZEB1 mRNA, promoting EMT and drug resistance	Cell-line and mechanistic evidence	Potential target for overcoming EMT-associated drug resistance	Ou et al. (2022)
m^6^A	YTHDF1	Reader	Hepatocellular carcinoma	Activates PI3K/AKT/mTOR signaling, promoting EMT and metastasis	Cell-line and mechanistic evidence	Potential pro-metastatic biomarker	Luo et al. (2021)
m^6^A	YTHDF1/DUSP5	Reader–target axis	Lung adenocarcinoma	Enhances DUSP5 translation, activates TGF-β/SMAD signaling, and drives EMT	Cell-line and mechanistic evidence	Potential target for EMT suppression	Fan et al. (2024)
m^6^A	YTHDF1	Reader	Laryngeal and breast cancers	Promotes translation of EIF4A3 and FOXM1, enhancing migration, invasion, and EMT	Cell-line evidence	May contribute to EMT and therapy resistance	Guo H. et al. (2024)
m^6^A	YTHDF2/NFIB	Reader–target axis	AR-negative castration-resistant prostate cancer	Stabilizes NFIB mRNA, promoting EMT and metastasis; cooperates with ALKBH5/TRIM8 signaling	Cell-line and mechanistic evidence	Potential target in aggressive prostate cancer	Shu et al. (2024)
m^6^A	YTHDF2/AXIN1	Reader–target axis	Cervical cancer	Regulates AXIN1 and modulates Wnt/β-catenin signaling, affecting EMT and chemoresistance	Cell-line and mechanistic evidence	May influence chemotherapy response	Wu et al. (2022)
m^6^A	YTHDF3/EGFR	Reader–target axis	Hepatocellular carcinoma	Enhances EGFR mRNA stability and activates EGFR/STAT3 signaling, promoting EMT	Cell-line and mechanistic evidence	Potential target for EGFR/STAT3-driven EMT	Chang et al. (2020)
m^6^A	YTHDC1/SMAD3	Nuclear reader	Triple-negative breast cancer	Promotes SMAD3 mRNA nuclear export, activating TGF-β signaling and EMT	Cell-line and mechanistic evidence	Potential target in TGF-β-driven EMT	Tan et al. (2022)
m^6^A	YTHDC1/LINC00641/HuR	Nuclear reader/lncRNA axis	Lung cancer	Regulates LINC00641 stability and indirectly enhances N-cadherin through HuR, promoting EMT and invasion	Cell-line evidence	Links EMT with ferroptosis sensitivity	Xi et al. (2023)
m^6^A	YTHDC2	Reader	Breast cancer	Maintains sphere formation, migration, and EMT marker expression	Cell-line evidence	Potential target for EMT-associated stemness	Tanabe et al. (2022)
m^6^A	HNRNPA2B1	m^6^A-associated reader	OSCC	Promotes EMT through the LINE-1/TGF-β/Smad/Slug pathway	Cell-line and mechanistic evidence	Potential EMT-related biomarker	Zhu et al. (2021)
m^6^A	HNRNPA2B1/MIR100HG	Reader/lncRNA axis	Colorectal cancer	Stabilizes TCF7L2 mRNA, activates Wnt/β-catenin signaling, and promotes EMT and drug resistance	Cell-line and mechanistic evidence	May contribute to drug resistance	Liu Z. et al. (2022)
m^5^C	NSUN2/YBX1	Writer–reader axis	Hepatocellular carcinoma	m^5^C modification of SREBP2 mRNA; YBX1 promotes mRNA stability, cholesterol biosynthesis, migration, and EMT	Cell-line and mechanistic evidence	Potential metabolic–EMT therapeutic target	Li K. J. et al. (2024)
m^5^C	NSUN2/ALYREF	Writer–reader axis	PM2.5-induced pulmonary fibrosis model	m^5^C modification of CHI3L1 mRNA; ALYREF promotes nuclear export and EMT-like fibrosis	Disease model evidence	Relevant to fibrosis-associated EMT	Shao et al. (2025)
m^5^C	ALYREF/EGFR	Reader–target axis	Hepatocellular carcinoma	Binds m^5^C sites in EGFR 3′UTR, stabilizes EGFR mRNA, activates STAT3 signaling, and promotes EMT	Cell-line and mechanistic evidence	Potential EGFR/STAT3-related target	Wang et al. (2023)
m^5^C	NSUN2/ALYREF/EZH2	Writer–reader–target axis	Cancer context not limited to one tumor type	Stabilizes EZH2 mRNA, activating EMT transcriptional programs	Mainly mechanistic evidence	Potential epigenetic–epitranscriptomic target	Wu et al. (2024a)
m^5^C	NSUN2/UBE2S	Writer–target axis	Lung adenocarcinoma	m^5^C modification of UBE2S, activating β-catenin signaling and enhancing EMT and metastasis	Cell-line and mechanistic evidence	Potential metastasis-related target	Chen Y. et al. (2025)
m^5^C	NSUN6/NM23-H1	Writer–target axis	Lung cancer	Stabilizes NM23-H1 mRNA and suppresses migration and EMT	Cell-line evidence	Tumor-suppressive m^5^C regulator	Lu et al. (2024)
m^5^C	MZF1-AS1/NSUN2/RAB13	lncRNA–writer–target axis	Gastric cancer peritoneal metastasis	Enhances m^5^C modification of RAB13 mRNA, increasing stability and promoting proliferation, invasion, EMT, and stemness	Cell-line and metastasis-related evidence	Potential target for peritoneal metastasis	Zhang W. et al. (2026)
m^5^C	NSUN2/ALYREF/YBX1/YAP	Writer–reader–target axis	NSCLC	Increases YAP mRNA stability and translation; activates CTGF, Cyr61, MMP2, and MMP9, promoting EMT	Cell-line and mechanistic evidence	Potential YAP-related therapeutic target	Li Y. et al. (2026)
m^5^C	NSUN2/PSD4	Writer–target axis	Breast cancer brain metastasis	Enhances PSD4 mRNA stability and export, promoting EMT, vascular mimicry, invasion, and ferroptosis resistance	Cell-line and metastasis model evidence	May contribute to brain metastasis and therapy resistance	Li M. et al. (2025)
A-to-I editing	ADAR1/GLI1	RNA-editing enzyme/target axis	Pancreatic ductal adenocarcinoma	circNEIL3 sponges miR-432-5p, increases ADAR1; ADAR1 edits GLI1 RNA, promoting cell cycle progression and EMT	Cell-line and clinical association evidence	Potential prognostic and therapeutic axis	Shen et al. (2021)
A-to-I editing	Edited miR-154-p13-5p	Edited miRNA	Bladder cancer	Edited miRNA targets LIX1L, suppressing proliferation, migration, and EMT while promoting apoptosis	Cell-line and clinical prognosis association	Potential tumor-suppressive RNA-editing biomarker	Hu et al. (2024)
A-to-I editing	ADAR1/Dicer	RNA-editing enzyme/miRNA-processing axis	OSCC	ADAR1 interacts with Dicer, enhances oncogenic miRNA production, and promotes EMT, invasion, migration, and cancer stemness	Cell-line and *in vivo* evidence; clinical prognosis association	Potential target for EMT and stemness inhibition	Yu et al. (2022)
ac^4^C	NAT10/HMGA1	Writer–target axis	Prostate cancer	Acetylates and stabilizes HMGA1 mRNA, promoting cell cycle progression	Cell-line and clinical correlation evidence	NAT10 may serve as tumor progression biomarker	Li R. et al. (2024)
ac^4^C	NAT10/KRT8	Writer–target axis	Prostate cancer	Stabilizes KRT8 mRNA, driving EMT, migration, and invasion	Cell-line and clinical association evidence	NAT10 inhibition may suppress EMT	Li R. et al. (2024)
ac^4^C	NAT10/TGFB1	Writer–target axis	Endometriosis/fibrosis models	Stabilizes TGFB1 mRNA, activates TGF-β signaling, and promotes fibrosis-associated EMT	Cell-line and disease model evidence	Remodelin reduces lesion growth and EMT-like fibrosis	Liu et al. (2025)
ac^4^C	NAT10	Writer	Gastric cancer	Activates Wnt/β-catenin and AKT signaling; promotes proliferation, migration, invasion, EMT, and chemotherapy resistance	Cell-line evidence	NAT10 inhibition may improve chemotherapy response	Jin et al. (2022)
ac^4^C	NAT10	Writer	NSCLC	NAT10 overexpression correlates with advanced T stage, lymph node metastasis, and poor survival; knockdown increases E-cadherin and decreases N-cadherin/vimentin	Cell-line and clinical correlation evidence	Remodelin may reverse EMT	Guo Q. et al. (2024)
ac^4^C	NAT10	Writer	Breast cancer	Promotes EMT-associated doxorubicin resistance; inhibition reverses EMT and restores drug sensitivity	Cell-line drug-resistance evidence	Potential target for overcoming doxorubicin resistance	Shenshen et al. (2023)
ac^4^C	NAT10	Writer	Hepatocellular carcinoma	Promotes invasion and migration through EMT activation, with reduced E-cadherin and increased vimentin	Cell-line evidence	Potential anti-metastatic target	Ma et al. (2016)
m^7^G	METTL1/WDR4	Writer complex	Nasopharyngeal carcinoma	Enhances m^7^G tRNA modification and codon-specific translation; activates Wnt/β-catenin signaling, promoting EMT, metastasis, and chemoresistance	Cell-line, mechanistic, and clinical association evidence	Potential target for translational reprogramming and chemoresistance	Chen X. et al. (2025)
m^7^G	WDR4/ZFAS1/RBFOX2	Writer/lncRNA feedback axis	Laryngeal cancer	Stabilizes ZFAS1 RNA, increases RBFOX2, decreases E-cadherin, and increases N-cadherin/vimentin	Cell-line and clinical association evidence	Potential prognostic and EMT-related target	Lu et al. (2025)
m^7^G	MYC/WDR4/CCNB1	Oncogene–writer–target axis	Hepatocellular carcinoma	MYC activates WDR4; WDR4 stabilizes CCNB1 mRNA, activates PI3K/AKT, suppresses p53, and promotes metastasis and sorafenib resistance	Cell-line and mechanistic evidence	Potential target for sorafenib resistance	Xia et al. (2021)
m^7^G-related translation	CELF1/eIF4E/PABPC1	Translation-regulatory complex	Breast epithelial/breast cancer models	Promotes non-canonical translation initiation and selective translation of EMT-related genes, enhancing dedifferentiation and migration	Cell-line and mechanistic evidence	Emerging translational target	Chaudhury et al. (2026)
Ψ	DKC1/ZFHX2-AS1/ARHGAP5	Pseudouridine synthase/lncRNA-target axis	Ovarian cancer	lncRNA-ZFHX2-AS1 suppresses DKC1-mediated pseudouridylation, reduces ARHGAP5 mRNA stability, inhibits Rho GTPase signaling, and suppresses EMT	Cell-line and mechanistic evidence	Potential EMT-suppressive regulatory axis	Dong et al. (2024)
Ψ	HSP90/PUS7/THUMPD1	Pseudouridylation-related axis	Gastric cancer	Promotes proliferation, migration, angiogenesis, chemoresistance, and EMT-associated progression	Cell-line and mechanistic evidence	Potential target for EMT and chemoresistance	Ye et al. (2025)

### 4.1 m^6^A-mediated EMT regulation

#### 4.1.1 m^6^A writers in EMT

m^6^A writers regulate EMT mainly by controlling the stability and translation of transcripts involved in signaling and cell-state transitions (Song and Zhou, 2021). METTL3 is the most frequently reported pro-EMT writer. In bladder cancer, METTL3 stabilizes P3H4 mRNA and promotes proliferation, migration, and EMT (Liu C. H. et al., 2024). In hepatocellular carcinoma, METTL3 methylates IQGAP3 mRNA, which is recognized by IGF2BP2. This increases IQGAP3 stability and activates TGF-β/SMAD signaling, leading to EMT and metastasis (Pan et al., 2025). In pancreatic cancer, METTL3 stabilizes AREG mRNA and promotes EMT, while miR-33a-3p negatively regulates this axis (Su et al., 2023).

METTL3 can also act as a tumor suppressor in certain cancer contexts. In renal cell carcinoma, METTL3 expression was found to be lower in tumor tissues than in adjacent non-tumor tissues, and higher METTL3 expression was associated with better patient survival (Li et al., 2017). Mechanistically, METTL3 may inhibit tumor growth by inducing G1-phase cell cycle arrest (Li et al., 2017). In glioblastoma, METTL3-mediated m6A modification restricts the self-renewal capacity of glioblastoma stem cells, whereas METTL3 knockdown promotes tumor progression and shortens survival in animal models (Cui et al., 2017). Similarly, in colorectal cancer, METTL3 suppresses cell proliferation, migration, and invasion through modulation of the p38/ERK signaling pathways and is associated with longer survival (De et al., 2019). In addition, reduced METTL3 expression can lead to global m6A hypomethylation in several cancers, thereby contributing to tumor progression. For example, in ocular melanoma, METTL3 downregulation, together with ALKBH5 upregulation, decreases m6A levels and is associated with earlier recurrence and increased tumor aggressiveness. METTL3-mediated m6A modification promotes the translation of the tumor suppressor HINT2 through a YTHDF1-dependent mechanism (Jia et al., 2019). In endometrial cancer, m6A hypomethylation caused by reduced METTL3 expression or METTL14 mutation disrupts AKT pathway regulation by downregulating the negative AKT regulator PHLPP2 and upregulating the positive AKT regulator mTORC2 (Liu J. et al., 2018). Overall, these findings indicate that METTL3 has context-dependent roles in cancer. In specific tumor types, METTL3 functions as a tumor suppressor by maintaining m6A homeostasis, enhancing tumor-suppressive transcript translation, inducing cell cycle arrest, suppressing cancer stemness, and inhibiting oncogenic signaling pathways. Therefore, METTL3 should not be universally described as an oncogene; its biological function depends on cancer type, cellular state, downstream m6A-modified transcripts, and the availability of m6A reader proteins.

The effect of METTL3 depends on cell type, disease state, reader availability, and target transcript selection. Therefore, the role of METTL3 in EMT should be interpreted in a context-dependent manner, considering tumor type, target transcripts, reader proteins, and the surrounding signaling environment. These contradictory findings suggest that RNA-modifying enzymes do not determine EMT outcomes by themselves. Instead, their biological effects are shaped by several context-dependent factors, including the modified transcript, the reader protein that recognizes the modification, the abundance of downstream signaling molecules, and the cellular or tumor-specific environment. For example, the same m6A writer may promote EMT when it stabilizes pro-EMT transcripts through IGF2BP proteins, but may suppress EMT if it enhances the degradation or translational inhibition of EMT-promoting targets through other reader-dependent mechanisms. Therefore, the functional outcome of RNA modification depends on an integrated enzyme–reader–target transcript–signaling context rather than on the expression level of the modifying enzyme alone.

In cervical cancer, METTL3-mediated m^6^A modification of KRAS enhances interactions with SMAD2/3 and SNAIL, activating the TGF-β/SMAD/SNAIL axis and extracellular matrix remodeling (Gong et al., 2026). These findings show that m^6^A does not simply regulate single genes. It can organize broader EMT networks through coding RNAs, circular RNAs, and signaling hubs. METTL14 is even more context dependent. In several cancers, it appears to suppress EMT by promoting mRNA decay. In bladder cancer, METTL14 inhibits migration and invasion by regulating USP38 through YTHDF2-mediated decay (Huang et al., 2022). In clear cell renal cell carcinoma, METTL14 promotes ITGB4 mRNA degradation and suppresses PI3K/AKT signaling, thereby reducing EMT (Liu Z. et al., 2022). However, in cervical cancer, METTL14 promotes EMT by methylating HOXB13 and activating NF-κB signaling (Li Q. et al., 2024). Thus, METTL14 can function as either a suppressor or promoter of EMT.

Non-canonical writers also contribute to EMT. RBM15 suppresses EMT in gastric cancer by regulating m^6^A modification of ECT2 mRNA through IGF2BP3 recognition and increases chemotherapy sensitivity (Zhu et al., 2026). In contrast, RBM15 stabilizes lncRNA HEIH in cervical cancer and promotes EMT through the miR-802/EGFR axis (Quan et al., 2024). METTL16 may regulate EMT indirectly by targeting genes such as FBXO5 and SYNPO2L, influencing PI3K/AKT signaling and tumor microenvironment remodeling (Wang et al., 2024; Wu J. et al., 2024). Overall, m^6^A writers regulate EMT through RNA stability, signaling activation, and non-coding RNA circuits. METTL3 is often pro-EMT in cancer, while METTL14, RBM15, and METTL16 show stronger context dependence. The key point is that writer expression alone is not enough to predict EMT outcome. The final effect depends on which transcripts are modified and which readers are present.

#### 4.1.2 m^6^A demethylases in EMT

ALKBH5 and FTO remove m^6^A marks and can reshape EMT programs by changing RNA stability and translation. Their roles are highly context dependent. ALKBH5 often suppresses EMT in cancer models. In NSCLC, ALKBH5 demethylates TGFβR2 and SMAD3 transcripts, reduces their stability, and inhibits TGF-β/SMAD signaling (Sun et al., 2022). It also upregulates SMAD6, which further suppresses EMT and invasion. In cisplatin-resistant models, ALKBH5 reduces EMT by regulating CEMIP mRNA stability and enhances drug sensitivity (Gao et al., 2024). These findings suggest that ALKBH5 can oppose EMT when it destabilizes pro-EMT transcripts. However, ALKBH5 can also promote EMT and stemness. In ovarian cancer, follicle-stimulating hormone activates the CREB/ALKBH5 axis, which reduces m^6^A modification on Snail mRNA, increases Snail stability, and promotes EMT and metastasis (Xu et al., 2024). ALKBH5 also regulates FOXP2 and CEP55, which may influence EMT progression (Yu et al., 2024). In NSCLC cancer stem cells, ALKBH5 supports stemness maintenance; its knockdown increases E-cadherin and decreases Nanog and Oct4 (Liu X. et al., 2022). These examples show that ALKBH5 can either inhibit or support EMT depending on the target transcript. The apparently opposite roles of ALKBH5 in EMT are not necessarily contradictory, but rather reflect its transcript- and context-dependent mode of action. In NSCLC, ALKBH5 demethylates TGFβR2 and SMAD3 transcripts, reduces their stability, attenuates TGF-β/SMAD signaling, and suppresses EMT (Sun et al., 2022). ALKBH5 also upregulates SMAD6, which further inhibits EMT and invasion. In contrast, in ovarian cancer, follicle-stimulating hormone activates the CREB/ALKBH5 axis, leading to reduced m^6^A modification on Snail mRNA, increased Snail stability, and enhanced EMT and metastasis (Xu et al., 2024). These divergent effects may be explained by differences in ALKBH5 target selection, upstream signaling inputs, and the cellular m^6^A reader landscape. For example, removal of m^6^A may stabilize a transcript when the mark normally recruits decay-promoting readers such as YTHDF2, but may destabilize a transcript when m^6^A-dependent stabilization is mediated by IGF2BP proteins. In addition, tissue-specific RNA-binding proteins, alternative splicing patterns, 3′UTR usage, and subcellular RNA localization may further determine whether ALKBH5 suppresses or promotes EMT in a given cancer context. Therefore, ALKBH5 should not be viewed as a universal EMT promoter or suppressor, but as a context-dependent regulator whose functional.

FTO shows a similar dual pattern. In thyroid cancer, FTO demethylates CDH12 mRNA and blocks IGF2BP2-mediated stabilization, thereby suppressing EMT (Chen Z. et al., 2024). In prostate cancer, FTO loss enhances EMT and migration, while FTO restoration reverses these effects (Zhao et al., 2024). In these settings, FTO appears to restrain EMT. In contrast, FTO promotes EMT in pancreatic cancer and breast cancer. In breast cancer, FTO stabilizes ZEB1 mRNA, supporting EMT and drug resistance (Ou et al., 2022). These contradictions should not be viewed as simple inconsistency. They reflect a core feature of epitranscriptomic regulation: the same enzyme can affect different RNA targets in different cells. Therefore, therapeutic inhibition of FTO or ALKBH5 may have different consequences across tumor types. Patient selection and transcript-level biomarkers will be essential. The context-dependent effects of FTO may be explained by differences in its dominant RNA targets, upstream signaling cues, and m^6^A reader landscape across cancer types. In thyroid and prostate cancers, FTO may preferentially demethylate transcripts that sustain epithelial identity or suppress EMT-related signaling, thereby reducing migration and invasion. In contrast, in breast and pancreatic cancers, FTO can act on pro-metastatic transcripts and enhance the stability or translation of EMT drivers, leading to increased invasion and metastasis. These divergent outcomes may also depend on the relative abundance of reader proteins such as YTHDF2 and IGF2BPs, because removal of m^6^A can either stabilize or destabilize a transcript depending on whether the original mark promotes RNA decay or supports RNA stability. In addition, lineage-specific RNA-binding proteins, alternative splicing patterns, 3′UTR usage, and subcellular localization of target RNAs may further shape the functional output of FTO. Therefore, FTO should not be simply classified as either an EMT promoter or suppressor; rather, its role reflects a transcript-specific and cellular-context-dependent regulatory mechanism.

#### 4.1.3 m^6^A reader proteins in EMT

Reader proteins are the execution layer of m^6^A signaling. They determine whether an m^6^A-marked transcript is translated, degraded, exported, or stabilized (Wen et al., 2025). YTHDF1 generally promotes EMT by enhancing translation of oncogenic transcripts. In hepatocellular carcinoma, YTHDF1 activates PI3K/AKT/mTOR signaling and promotes EMT and metastasis (Luo et al., 2021). In lung adenocarcinoma, YTHDF1 enhances DUSP5 translation and activates TGF-β/SMAD signaling (Fan et al., 2024). In laryngeal and breast cancers, it promotes translation of EIF4A3 and FOXM1, increasing migration, invasion, and EMT (Guo H. et al., 2024). These findings support YTHDF1 as a pro-translation and pro-EMT reader. YTHDF2 is more complex. It is often considered a decay-promoting reader, but its functional outcome depends on which transcript is degraded. In androgen receptor-negative castration-resistant prostate cancer, YTHDF2 stabilizes NFIB mRNA and promotes EMT and metastasis (Shu et al., 2024). It also cooperates with ALKBH5/TRIM8 signaling to enhance NFIB protein stability (Shu et al., 2024). In cervical cancer, YTHDF2 regulates AXIN1 and Wnt/β-catenin signaling, thereby influencing EMT and chemoresistance (Wu et al., 2022). Thus, YTHDF2 cannot be simply defined as tumor suppressive or oncogenic. YTHDF3 promotes EMT in several models. In hepatocellular carcinoma, it stabilizes EGFR mRNA and activates EGFR/STAT3 signaling (Chang et al., 2020). In breast cancer, YTHDF3 enhances translation of Notch2, ZEB1, and SMAD5, promoting EMT and bone metastasis (Liu Z. et al., 2022). YTHDC1 functions mainly in the nucleus (Chang et al., 2020). In triple-negative breast cancer, YTHDC1 promotes SMAD3 mRNA export and activates TGF-β signaling (Tan et al., 2022). YTHDC2 has been linked to breast cancer stemness, migration, and EMT marker expression, although its mechanisms vary by subtype (Tanabe et al., 2022). Other readers and reader-like proteins also participate in EMT. HNRNPA2B1 promotes EMT in oral squamous cell carcinoma through the LINE-1/TGF-β/Smad/Slug pathway (Zhu et al., 2021). In colorectal cancer, it stabilizes TCF7L2 mRNA with MIR100HG and activates Wnt/β-catenin signaling, forming a feedback loop that enhances EMT and drug resistance (Liu H. et al., 2022). Overall, m^6^A readers translate the presence of an RNA mark into a biological outcome. YTHDF1 and YTHDF3 are often pro-EMT, YTHDC1 links nuclear RNA processing to EMT, and YTHDF2 shows strong context dependence. Future studies should move beyond measuring total m^6^A and instead define which reader binds which modified transcript in each EMT state.

### 4.2 m^5^C in EMT

m^5^C regulates EMT mainly through transcript stabilization, nuclear export, and translation. NSUN2 is the most studied m^5^C writer in cancer (Huang et al., 2024). It often cooperates with ALYREF or YBX1 to increase expression of pro-metastatic transcripts. In HCC, ALYREF binds m^5^C sites in the EGFR 3′UTR, stabilizes EGFR mRNA, and activates STAT3 signaling (Wang et al., 2023). The NSUN2–ALYREF axis also stabilizes EZH2 mRNA and activates EMT transcriptional programs (Wu et al., 2024a). In NSCLC, NSUN2 enhances m^5^C modification of YAP mRNA, and ALYREF/YBX1 increase YAP stability and translation. This activates CTGF, Cyr61, MMP2, and MMP9, leading to EMT and tumor progression (Li R. et al., 2026). In breast cancer brain metastasis, NSUN2 modifies PSD4 mRNA, promoting stability, export, EMT, vascular mimicry, and ferroptosis resistance (Li M. et al., 2025). These studies suggest that NSUN2 is a central pro-EMT m^5^C writer. However, this conclusion must be balanced by evidence that NSUN6 can suppress migration and EMT in lung cancer by stabilizing NM23-H1 mRNA (Zhao et al., 2024). Thus, m^5^C is not inherently pro-metastatic. The tumor-promoting pattern mainly reflects the dominant role of NSUN2 in available studies. A limitation of the current m^5^C literature is that many studies show associations among NSUN2, m^5^C, and target RNA stability, but fewer demonstrate site-specific causality. Future work should validate individual m^5^C sites and test whether mutating those sites is sufficient to block EMT.

### 4.3 Role of A-to-I RNA editing in EMT

A-to-I RNA editing regulates EMT by altering RNA sequence, miRNA processing, circular RNA networks, and innate immune sensing (Liang et al., 2024). ADAR1 is the major cancer-associated enzyme (Shen et al., 2021). In pancreatic ductal adenocarcinoma, circNEIL3 sponges miR-432-5p and increases ADAR1 expression. ADAR1 edits GLI1 RNA, promoting cell-cycle progression and EMT (Shen et al., 2021). This axis is associated with clinical stage and survival. A feedback loop also exists, in which ADAR1 regulates circNEIL3 biogenesis. This suggests that editing enzymes can participate in self-reinforcing RNA networks. In bladder cancer, edited miR-154-p13-5p appears to have tumor-suppressive activity. High expression is associated with better prognosis, and the edited miRNA targets LIX1L to suppress proliferation, migration, and EMT while promoting apoptosis (Hu et al., 2024). In oral squamous cell carcinoma, ADAR1 is upregulated and associated with poor prognosis. It interacts with Dicer and enhances oncogenic microRNA production, thereby promoting migration, invasion, EMT, and cancer stemness (Yu et al., 2022). These examples show that A-to-I editing has dual effects. ADAR1 often promotes EMT and immune escape, but specific edited RNAs may suppress tumor progression. This duality is important for therapy. Global ADAR1 inhibition may enhance anti-tumor immunity, but it could also remove protective editing events in some contexts. Therefore, the most useful biomarkers may not be total ADAR1 expression, but specific editing signatures linked to EMT and immune resistance.

### 4.4 ac4C in EMT

ac4C is mainly mediated by NAT10 and regulates EMT by increasing mRNA stability and translation efficiency (Xiong et al., 2026). NAT10 has been linked to EMT in several cancers. In prostate cancer, NAT10 is upregulated and associated with tumor grade, stage, and Gleason score. It acetylates HMGA1 mRNA and increases its stability, promoting cell-cycle progression. NAT10 also stabilizes KRT8 mRNA, driving EMT and enhancing migration and invasion (Li K. J. et al., 2024). In gastric cancer, NAT10 promotes proliferation, migration, invasion, chemotherapy resistance, AKT phosphorylation, and EMT marker changes through Wnt/β-catenin signaling (Jin et al., 2022). In NSCLC, NAT10 expression is associated with advanced T stage, lymph node metastasis, and poor survival. NAT10 knockdown increases E-cadherin and decreases N-cadherin and vimentin, while Remodelin reverses EMT-associated change (Guo Q. et al., 2024). In breast cancer, NAT10 contributes to doxorubicin resistance by maintaining EMT, and its inhibition restores drug sensitivity (Shenshen et al., 2023). In hepatocellular carcinoma, NAT10 promotes migration and invasion with reduced E-cadherin and increased vimentin (Ma et al., 2016). However, important gaps remain. NAT10 has functions beyond RNA acetylation, and ac4C readers and erasers are not well defined. Therefore, some observed effects of NAT10 inhibition may not be entirely ac4C dependent. More precise tools are needed to separate RNA-acetylation-dependent effects from other NAT10 functions. It should also be noted that NAT10 is a multifunctional enzyme, and its biological effects are not limited to mRNA ac^4^C deposition. In addition to acetylating RNA, NAT10 has been implicated in ribosomal RNA processing, ribosome biogenesis, nucleolar function, nuclear architecture, and chromatin-associated regulation, including potential effects on histone acetylation. Therefore, phenotypes caused by global NAT10 inhibition may not solely reflect loss of ac^4^C on specific EMT-related transcripts. Remodelin-mediated NAT10 inhibition may alter EMT through combined effects on RNA stability, ribosome production, translational capacity, nuclear organization, and chromatin state. This multifunctionality should be considered when interpreting NAT10-targeting studies, especially because global inhibition may produce broader cellular stress responses that indirectly influence EMT, invasion, and therapeutic sensitivity.

### 4.5 m^7^G in EMT

m^7^G regulates EMT mainly through translational reprogramming. METTL1 and WDR4 catalyze m^7^G modification of tRNA and some mRNA sites, thereby affecting codon-specific translation and protein production (Gu et al., 2024; Li Y. et al., 2026). In nasopharyngeal carcinoma, METTL1 and WDR4 are upregulated and associated with poor prognosis. They increase tRNA m^7^G modification, reshape translation efficiency, activate Wnt/β-catenin signaling, and promote EMT, metastasis, and chemoresistance (Chen B. et al., 2022). ARNT further regulates METTL1 expression, linking environmental signaling to translational control. In laryngeal cancer, WDR4 stabilizes ZFAS1 RNA and increases RBFOX2 expression, forming a positive feedback loop that decreases E-cadherin and increases N-cadherin and vimentin (Lu et al., 2025). In hepatocellular carcinoma, MYC activates WDR4, which stabilizes CCNB1 mRNA, promotes G2/M transition, activates PI3K/AKT signaling, suppresses p53, and enhances metastasis and sorafenib resistance (Xia et al., 2021). m^7^G-related EMT may also involve non-canonical translation. In breast epithelial cells, CELF1 interacts with eIF4E and PABPC1 to promote non-canonical translation initiation, selectively increasing translation of EMT-related genes and supporting dedifferentiation and migration (Chaudhury et al., 2026). The m^7^G field is important because it shifts attention from transcript abundance to translation bias. EMT requires rapid production of proteins involved in cytoskeletal remodeling, migration, and survival. m^7^G may support this by tuning the translation machinery. However, direct mapping of m^7^G-modified EMT transcripts remains limited, and more mechanistic studies are needed. Although METTL1-mediated tRNA m^7^G modification has been linked to codon-specific translational control, whether EMT programs rely on a defined codon bias remains unclear. One possibility is that EMT-associated transcripts, including those encoding Snail, vimentin, ZEB1, and extracellular matrix proteins, may preferentially use codons decoded by m^7^G-modified tRNAs, thereby gaining a translational advantage during EMT. In this model, METTL1 would not simply increase global protein synthesis, but would reshape the EMT proteome by enhancing the translation of codon-matched mesenchymal transcripts. Nevertheless, direct evidence identifying specific codons or amino acid-specific tRNA sets enriched during EMT is still limited. Further ribosome profiling and tRNA modification studies are required to clarify whether METTL1 exploits an EMT-associated codon usage pattern.

### 4.6 Role of Ψ modification in EMT

Ψ is catalyzed by pseudouridine synthases and H/ACA ribonucleoprotein complexes (Wu et al., 2024b). It contributes to RNA stability, ribosome function, translation fidelity, and stress adaptation. Evidence linking Ψ to EMT is still emerging. In gastric cancer, the HSP90/PUS7/THUMPD1 axis promotes proliferation, migration, angiogenesis, chemoresistance, and EMT-related progression (Ye et al., 2025). These studies suggest that Ψ-related enzymes may either support or restrain EMT depending on their target RNAs and interacting partners. At present, Ψ should be considered an underexplored EMT regulator. The field needs better transcriptome-wide mapping, site-specific validation, and functional models to determine whether Ψ is a driver of EMT or mainly a marker of broader RNA and ribosome remodeling.

RNA modifications may also contribute to partial or hybrid EMT states rather than only regulating complete EMT. Partial EMT cells retain some epithelial features while acquiring mesenchymal traits, which can enhance collective migration, metastatic plasticity, stemness, and therapy resistance (Lüönd et al., 2021). Because RNA modifications regulate transcript stability, translation, splicing, and non-coding RNA networks, they may fine-tune the expression of EMT-related genes instead of simply switching epithelial markers off and mesenchymal markers on. For example, context-dependent m6A regulation of transcripts involved in TGF-β, Wnt/β-catenin, hypoxia, and inflammatory signaling may generate intermediate EMT phenotypes (Pan et al., 2025; Liu H. et al., 2022). Single-cell transcriptomic studies further suggest that EMT programs are heterogeneous within tumors, supporting the possibility that RNA modification enzymes regulate distinct epithelial, mesenchymal, and hybrid EMT cell populations (Puram et al., 2017). Therefore, future studies combining single-cell analysis with site-specific epitranscriptomic mapping will be important to define how RNA modifications control partial EMT dynamics during metastasis and therapeutic resistance.

### 4.7 Non-coding RNA–Epitranscriptomic feedback networks in EMT plasticity

Beyond individual examples such as m^6^A-modified HEIH, m^5^C-regulated MZF1-AS1, circPSMA7, LINC00618, and circNEIL3, non-coding RNAs should be considered as dynamic regulatory nodes within epitranscriptomic networks rather than isolated downstream targets. Recent evidence suggests that lncRNAs and circRNAs can regulate EMT through multiple mechanisms, including recruiting RNA-modifying enzymes to specific transcripts, modulating the stability or activity of these enzymes, acting as competing endogenous RNAs, and reshaping signaling pathways related to β-catenin, Rho GTPase, MAPK, cholesterol metabolism, and ADAR1-mediated RNA editing (Li R. et al., 2024; Shen et al., 2021; Eladl and Estfanous, 2026; Chen Y. et al., 2025; Zhang Q. et al., 2026; Dong et al., 2024; Yi et al., 2024; Xi et al., 2023). Importantly, the functions of non-coding RNAs are highly context-dependent and may be influenced by RNA structure, subcellular localization, phase-separation behavior, hypoxia, inflammation, metabolic stress, and therapeutic pressure (Eladl and Estfanous, 2026). RNA modifications can further enhance this plasticity by altering non-coding RNA processing, folding, stability, turnover, and interactions with RNA-binding proteins, whereas non-coding RNAs can in turn regulate the expression, recruitment, or activity of RNA-modifying enzymes. Therefore, non-coding RNA–RNA modification interactions form reciprocal feedback circuits that help cancer cells integrate microenvironmental stress with EMT plasticity, stemness, metabolic adaptation, invasion, and metastatic progression.

## 5 Crosstalk with the tumor microenvironment

**FIGURE 3:**
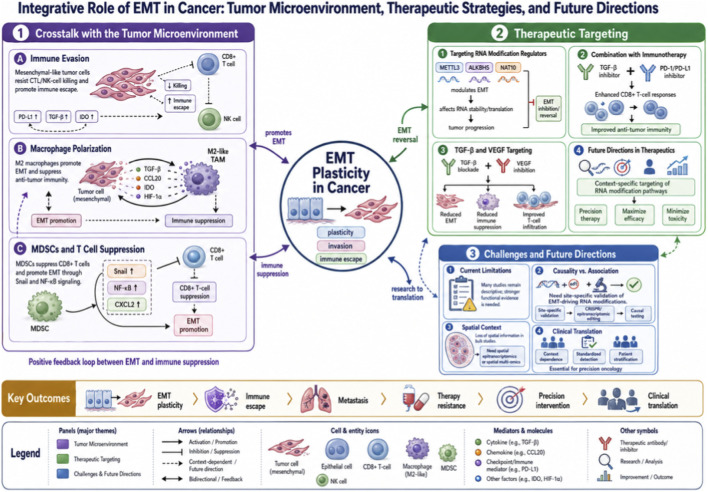
Integration of EMT plasticity with the tumor microenvironment, therapeutic targeting, and future directions. This figure summarizes how EMT plasticity interacts with the tumor microenvironment and contributes to immune escape, macrophage polarization, myeloid-derived suppressor cell-mediated T cell suppression, metastasis, and therapy resistance. EMT programs can promote immune evasion by increasing inhibitory mediators such as PD-L1, TGF-β, and IDO, while M2-like tumor-associated macrophages and MDSCs further reinforce EMT and immunosuppression. Therapeutically, targeting RNA modification regulators or combining EMT-related interventions with immunotherapy may help reverse EMT-associated immune suppression and improve anti-tumor responses. The key message is that RNA modification-driven EMT should be studied within its spatial and immune context, and future work should emphasize causal validation, spatial epitranscriptomics, patient stratification, and precision therapeutic intervention. Abbreviations: EMT, epithelial–mesenchymal transition; TME, tumor microenvironment; TAM, tumor-associated macrophage; MDSC, myeloid-derived suppressor cell; PD-L1, programmed death-ligand 1; IDO, indoleamine 2,3-dioxygenase; TGF-β, transforming growth factor-beta; VEGF, vascular endothelial growth factor; NK cell, natural killer cell.

Beyond tumor-cell-intrinsic regulation, EMT is closely connected with the tumor microenvironment and therapeutic response. We therefore summarize the reciprocal interaction among EMT, immune suppression, therapeutic targeting, and future translational challenges **(** Figure 3).

### 5.1 EMT-induced immune evasion and immune cell remodeling

The tumor microenvironment is shaped by hypoxia, acidity, inflammation, nutrient stress, stromal remodeling, and immune suppression (Imodoye and Adedokun, 2023). Tumor cells interact with macrophages, fibroblasts, MDSCs, T cells, B cells, adipocytes, mast cells, and endothelial cells through cytokines, chemokines, metabolites, and extracellular matrix signals (Imodoye and Adedokun, 2023). These interactions can induce EMT. In turn, EMT can make the tumor microenvironment more immunosuppressive (Singh and Chakrabarti, 2019). Tumor-associated macrophages are among the most important EMT-promoting immune cells. M2-like macrophages secrete TGF-β, COX-2-related mediators, EGFR/ERK activators, AKT/mTOR signals, CCL18, and hypoxia-related factors, all of which can support EMT (Singh and Chakrabarti, 2019). Under hypoxia, HIF-1α induces tumor cells to secrete CCL20, which increases IDO expression in macrophages and suppresses CD4^+^ and CD8^+^ T-cell function (Jin et al., 2018). This creates a link among hypoxia, macrophage polarization, EMT, and immune evasion. MDSCs also contribute to this loop. They suppress T cells, NK cells, and dendritic cells. In addition, Snail can activate NF-κB signaling and increase CXCL2 expression, thereby promoting MDSC infiltration and CD8^+^ T-cell suppression (Li Z. et al., 2024). MDSCs can also directly induce EMT, further enhancing invasion and metastasis (Zhang et al., 2022). EMT transcription factors themselves have immune-regulatory functions. Snail promotes Treg recruitment and tolerogenic dendritic cells. Twist suppresses inflammatory responses through TNF-α and NF-κB-related mechanisms and can increase Treg infiltration while reducing CD8^+^ T-cell and NK-cell activity. ZEB1 and ZEB2 impair dendritic cell maturation and cytotoxic T-cell activation (Zhang et al., 2022). Thus, EMT transcription factors are not only cell-intrinsic regulators of migration. They also remodel immune surveillance.

### 5.2 EMT, immune escape, and cancer cell plasticity

Mesenchymal-like tumor cells often resist cytotoxic T lymphocyte and NK-cell killing (Oh et al., 2018). In breast and lung cancer models, mesenchymal cells show reduced sensitivity to CTL-mediated cytotoxicity, partly through autophagy and TGF-β signaling (Yu et al., 2013). This provides a mechanism by which EMT supports immune escape. However, EMT does not always reduce immune recognition. In some models, EMT increases NK-cell-activating ligands such as NKG2DL and may make tumor cells more sensitive to NK-cell killing (Yu et al., 2013; Murakami et al., 2025). Changes in CADM1 and E-cadherin may also influence NK-cell recognition (Murakami et al., 2025). These findings show that EMT–immune interactions are not one-directional. The immune outcome depends on tumor type, EMT state, immune context, and timing. This nuance is important for therapy. Blocking EMT may not always enhance immunity in the same way. In some tumors, suppressing EMT may reduce immune escape. In others, targeting specific immunosuppressive signals produced by EMT-like cells may be more effective than broadly reversing EMT.

### 5.3 Immune checkpoints and EMT–TME feedback

TGF-β and VEGF are central mediators of EMT, angiogenesis, immune exclusion, and extracellular matrix remodeling. Dual targeting of TGF-β and VEGF can suppress EMT and immune inhibition and may enhance PD-1 blockade by increasing both the number and function of tumor-infiltrating lymphocytes (Zhang et al., 2025). TIM-3 may also contribute to EMT-associated immune escape. During early micrometastasis, TIM-3 is upregulated in tumor cells and promotes stemness, immune suppression, and TME remodeling through the β-catenin/IL-1β axis (Niu et al., 2023). This suppresses CD8^+^ T-cell function and promotes immunosuppressive γδ T-cell formation, helping micrometastases progress to macrometastases (Niu et al., 2023). Overall, EMT and immune evasion form a feedback loop. Immune and stromal signals such as TGF-β, NF-κB, HIF-1α, and COX-2 induce EMT. EMT then increases immunosuppressive signaling, changes immune-cell composition, and promotes checkpoint resistance (Ye et al., 2025). RNA modifications may strengthen this loop by rapidly adjusting the stability and translation of EMT and immune-regulatory transcripts.

## 6 Therapeutic targeting

### 6.1 Targeting RNA modification regulators

RNA modification enzymes are attractive therapeutic targets because they are enzymatic and potentially druggable. However, they also regulate many normal cellular processes. Therefore, therapeutic development must balance anti-tumor efficacy with toxicity and context dependence. METTL3 inhibition is one example. The METTL3 inhibitor STM2457, combined with the tyrosine kinase inhibitor anlotinib, suppresses oral squamous cell carcinoma growth, promotes apoptosis, and reduces stemness and EMT features (Yankova et al., 2021). Mechanistically, this effect is linked to EGFR downregulation, suggesting that EGFR may connect m^6^A inhibition with reduced EMT signaling. In ovarian cancer, METTL3 promotes progression by enhancing YTHDF2-dependent degradation of GATA4. METTL3 inhibition restores GATA4 expression and suppresses proliferation, invasion, and EMT (Chen X. et al., 2022). Single-cell analysis suggests that GATA4 loss is associated with EMT activation, proliferation, and extracellular matrix remodeling in multiple stromal and tumor compartments (Chen X. et al., 2022). ALKBH5 and FTO are also potential targets, but they require careful tumor-specific evaluation. In NSCLC cancer stem cells, ALKBH5 maintains stemness, and its knockdown increases E-cadherin while reducing Nanog and Oct4 (Liu X. et al., 2022). In contrast, ALKBH5 can suppress TGF-β/SMAD signaling in other contexts (Lüönd et al., 2021). FTO loss can promote EMT in prostate cancer by affecting Wnt-related transcripts and DDIT4 stabilization through IGF2BP2/3 (Zhao et al., 2024). Yet FTO inhibition by the small molecule C6 suppresses esophageal cancer growth through EMT and PI3K/AKT regulation (Qin et al., 2022). These examples show why global inhibition of demethylases may not be universally beneficial.

NAT10 is a promising target because many studies link it to ac4C-mediated EMT, migration, invasion, and drug resistance. Remodelin or NAT10 knockdown can suppress EMT in NSCLC and other cancer models (Guo Q. et al., 2024). In breast cancer, NAT10 supports chemotherapy resistance by maintaining EMT, and its inhibition restores E-cadherin and reduces vimentin (Wu et al., 2018). NSUN2 is another potential target because NSUN2-mediated m^5^C stabilizes YAP mRNA with ALYREF and YBX1 cooperation, promoting EMT and tumor progression (Liu D. et al., 2024). Overall, targeting METTL3, FTO, ALKBH5, NAT10, NSUN2, or related reader proteins may reverse EMT and reduce metastasis. However, the major challenge is selectivity. These enzymes regulate many transcripts. Future therapy should move toward context-specific targeting based on modified target RNAs, reader dependencies, and EMT state rather than enzyme expression alone.

### 6.2 Combination with immunotherapy

TGF-β signaling promotes EMT, extracellular matrix remodeling, immune exclusion, and T-cell dysfunction. Therefore, combining TGF-β inhibition with PD-1/PD-L1 blockade is a rational strategy (Niu et al., 2023). Galunisertib, a TGF-β receptor I inhibitor, has been tested with nivolumab in clinical trials involving pancreatic ductal adenocarcinoma, liver cancer, and NSCLC (NCT02734160, NCT02423343) (Melisi et al., 2021; Nadal et al., 2023). Preclinical studies show that galunisertib plus anti-PD-L1 therapy enhances tumor regression and T-cell responses compared with monotherapy. Other TGF-β-targeting strategies show similar logic. LY364947 increases CD8^+^ T-cell infiltration in colon adenocarcinoma models and improves anti-PD-L1 efficacy (Tschernia and Gulley, 2022). TGF-β-targeted oncolytic viruses also synergize with PD-1 blockade in breast and renal cancer models. M7824, a bifunctional fusion protein targeting PD-L1 and TGF-β, reverses EMT-like mesenchymalization and enhances CD8^+^ T-cell and NK-cell activity in preclinical models (GSK, 2019). Signaling inhibitors may also improve checkpoint therapy. MEK inhibition combined with PD-L1 blockade shows activity in several solid tumors, especially melanoma and NSCLC, and can increase CD8^+^ T-cell infiltration and MHC-I expression (Limagne et al., 2022). EGFR signaling is linked to immune escape because EGFR activation can upregulate PD-L1, while EGFR tyrosine kinase inhibitors can reduce PD-L1 and improve cytotoxic T-cell sensitivity (Rosell and Palmero, 2015). The key question is where RNA modification targeting fits into these combinations. One possibility is that RNA modification inhibitors could reduce EMT plasticity and improve immune recognition. Another is that they could reshape cytokine production, antigen presentation, or immune checkpoints. However, this remains underexplored. Future studies should test RNA modification inhibitors together with checkpoint blockade in immune-competent models rather than relying only on tumor-cell assays.

## 7 Challenges and future directions

Despite rapid progress, several major problems limit the field. First, many studies remain descriptive. They show that a writer, eraser, or reader is associated with EMT markers, migration, invasion, or prognosis. However, association is not causality. Stronger evidence requires site-specific validation. For example, if an m^6^A or m^5^C site on a target transcript is proposed to drive EMT, mutation or editing of that site should reduce the EMT phenotype. Without this, it is difficult to know whether the modification is a driver or a marker. Second, most current data come from bulk assays such as MeRIP-seq, m^6^A-seq, and standard RNA sequencing. These methods average signals across mixed cell populations. EMT is highly heterogeneous, and partial EMT cells may represent only a small fraction of the tumor. Bulk analysis can therefore miss the most important cell states. True single-cell epitranscriptomic methods are still technically difficult because of low RNA input, antibody limitations, signal dilution, and sequencing depth. Third, spatial information is missing. EMT often occurs at invasive fronts, hypoxic regions, perivascular niches, or immune-excluded areas. Bulk or dissociated single-cell assays lose this spatial context. Spatial epitranscriptomics is still at an early stage, but it will be essential for understanding how RNA modifications regulate EMT in specific tumor niches. Fourth, EMT measurement is often oversimplified. Many studies rely on E-cadherin, N-cadherin, and vimentin. These markers are useful but incomplete. EMT should be measured as a spectrum using marker panels, transcriptional trajectories, functional assays, and spatial localization. This is especially important for partial EMT and MET.

Fifth, clinical translation remains difficult. RNA modification levels are not yet standardized in clinical samples. Different platforms may produce different results. In addition, RNA modification regulators are context dependent. A molecule that promotes EMT in one tumor may suppress it in another. This limits the use of single enzymes as universal biomarkers or targets. Future work should integrate several approaches. Single-cell and spatial multi-omics can define which tumor and stromal cells carry specific RNA modification programs. CRISPR-based editing and site-specific epitranscriptomic tools can test causality. Live-cell imaging and lineage tracing can connect RNA modification dynamics to EMT transitions. Large clinical cohorts can determine whether specific modification–target–reader axes predict metastasis, immune resistance, or therapy response. Machine learning may help integrate these datasets and identify clinically useful EMT–epitranscriptomic signatures.

Emerging technologies will be essential for resolving the causal and spatial complexity of RNA modification-mediated EMT regulation. Single-cell and spatial epitranscriptomic approaches may help identify whether specific RNA modification enzymes or modified transcripts are enriched in epithelial, mesenchymal, or hybrid partial-EMT cell states within tumors (Puram et al., 2017). Direct RNA sequencing and long-read sequencing can further improve the detection of transcript isoforms, RNA modification patterns, and non-coding RNA regulation without relying solely on antibody-based enrichment methods (Kim et al., 2025). In addition, CRISPR-based epitranscriptomic editing tools may allow site-specific manipulation of RNA modifications (Yi et al., 2023), providing stronger causal evidence that individual modification sites directly regulate EMT-related transcripts and phenotypic plasticity.

## 8 Conclusion

RNA modifications form a reversible post-transcriptional layer that helps cancer cells regulate EMT. They do not replace transcription factors, signaling pathways, or chromatin regulation. Instead, they add speed, flexibility, and context sensitivity to these systems. Through effects on RNA stability, translation, splicing, export, immune sensing, and noncoding RNA function, RNA modifications connect hypoxia, TGF-β signaling, inflammation, metabolic stress, and therapy pressure to EMT plasticity. Noncoding RNAs should also be considered in this regulatory network, because lncRNAs, circRNAs, and miRNAs can serve as direct substrates of RNA modifications and, in turn, modulate the expression, localization, or activity of RNA-modifying enzymes. These bidirectional interactions create feedback loops that further shape EMT-associated signaling, cellular plasticity, immune escape, and therapeutic resistance. m^6^A has the strongest evidence and regulates EMT through writer–reader–eraser networks. m^5^C and ac^4^C mainly promote EMT by stabilizing and translating pro-metastatic transcripts. m^7^G supports EMT through translational reprogramming. A-to-I editing links EMT with RNA sequence diversity and innate immune evasion. Ψ is still less understood but may regulate EMT through RNA stability and stress adaptation. The field now needs to move beyond simple lists of regulators. The most important questions are which RNA modification sites are causal, which reader proteins execute their effects, which EMT states depend on them, which noncoding RNA circuits participate in these processes, and which patients may benefit from targeting these pathways. With better single-cell, spatial, and functional tools, RNA modifications and their associated noncoding RNA networks may become actionable targets for limiting EMT, metastasis, and therapy resistance in cancer.
